# OVOL1 Promotes Proliferation and Metastasis of Non‐Small Cell Lung Cancer by Regulating APOE‐Mediated Cholesterol Metabolism

**DOI:** 10.1111/jcmm.70634

**Published:** 2025-05-28

**Authors:** Shoujie Feng, Li Zhang, Teng Sun, Lei Xu, Xiaoyu Quan, Guoqing Zhao, Hao Zhang

**Affiliations:** ^1^ Thoracic Surgery Laboratory Xuzhou Medical University Xuzhou Jiangsu China; ^2^ Department of Thoracic Surgery Affiliated Hospital of Xuzhou Medical University Xuzhou Jiangsu China; ^3^ Editorial Office of International Journal of Anesthesiology and Resuscitation Xuzhou Medical University Xuzhou Jiangsu China; ^4^ Department of Emergency Medicine The Affiliated Hospital of Xuzhou Medical University Xuzhou Jiangsu China

**Keywords:** cholesterol, lung cancer, metabolism

## Abstract

Non‐small cell lung cancer (NSCLC) is a highly lethal malignant tumour characterised by its resistance to treatment, often due to metabolic reprogramming. Despite this, the underlying mechanisms by which aberrant cholesterol metabolism influences the development and progression of NSCLC remain unclear. In our study, we observed that OVOL1 is significantly upregulated in NSCLC and is correlated with a poor prognosis. Furthermore, our functional assays revealed that OVOL1 enhances the proliferation and metastasis of NSCLC cells both in vitro and in vivo. Mechanistically, OVOL1 was found to modulate cholesterol reprogramming and increase the expression of APOE, thereby intensifying cholesterol metabolism and facilitating cell migration and invasion. In conclusion, our findings suggest that OVOL1 acts as an oncogene in NSCLC, promoting tumour growth and metastasis through the enhancement of cholesterol metabolism. This underscores the potential of OVOL1 as a therapeutic target for the treatment of NSCLC.

## 1 Introduction

Lung cancer is one of the most serious malignant tumours, seriously threatening human health and quality of life [1]. In 2024, the most recent information from the International Agency for Research on Cancer shows that there were 2.481 million new instances of lung cancer diagnosed, accounting for 12.4% of the world's new cancer cases and becoming the world's largest cancer. Notably, lung cancer ranks first in cancer mortality for both men and women in China [2]. Simultaneously, lung cancer continues to be the primary reason for mortality, boasting an 18.7% mortality rate. Approximately 85% of all lung cancers are classified as non‐small cell lung cancer (NSCLC) [3, 4]. The management of lung cancer remains a significant clinical challenge, underscored by the limited success in improving long‐term survival rates for patients [5]. The quest for a deeper understanding of the biological mechanisms that drive NSCLC progression and metastasis is crucial for developing innovative therapeutic strategies.

Recent advances in cancer biology have elucidated the pivotal role that metabolic reprogramming plays in the oncogenesis and progression of malignancies [6]. Of various altered metabolic pathways, cholesterol metabolism has attracted considerable attention due to its complex role in forming cell membranes, producing steroid hormones and modulating cell signalling [7, 8, 9, 10, 11]. Specifically, aberrations in cholesterol metabolism, including heightened synthesis and deregulated uptake, are intimately linked to the pathogenesis of NSCLC [12, 13, 14, 15]. The augmentation of cholesterol biosynthesis equips lung cancer cells with enhanced survival capabilities, particularly post‐exposure to epidermal growth factor receptor tyrosine kinase inhibitors [16, 17]. The enzyme 3‐hydroxy‐3‐methylglutaryl‐CoA reductase (HMGCR), which is elevated in human NSCLC tissues, becomes a therapeutic target as its inhibition by fluvastatin curtails tumourigenesis of NSCLC by abating tumour proliferation and promoting apoptosis of cancer cells [18]. Meanwhile, 3‐hydroxy‐3‐methylglutaryl‐CoA synthase (HMGCS), catalysing the conversion of acetyl‐CoA to HMG‐CoA, is instrumental in the cholesterol synthesis pathway [19]. Cop9 signalosome 6 (CSN6) enhances the stability of the HMGCS1 protein by forestalling its ubiquitination and subsequent degradation, mediated by the SPOP protein [20]. Beyond the structural role, cholesterol and its derivatives also exert influence on the tumour immune microenvironment; for instance, oxysterols, the oxidative derivatives of cholesterol, can modulate transcription factors pertinent to cholesterol homeostasis (SREBP2 and LXR), culminating in T cell dysfunction [21]. Consequently, strategic reprogramming of cholesterol metabolism presents a promising avenue to regulate the progression of NSCLC.

Ovo like transcriptional repressor 1 (OVOL1), a zinc finger transcription factor, is a member of the OVOL protein family [22]. Its main functions include regulating cell differentiation and development. OVOL1 is integral to numerous biological processes, with its significance particularly pronounced in the development of skin and hair [22, 23, 24]. However, in the field of oncology, the expression and function of OVOL1 have also attracted the attention of researchers. Although OVOL1's impact on cancer advancement has been documented, its role in regulating cholesterol metabolism, especially in NSCLC, has not been well studied.

Our study demonstrated the role of OVOL1 in NSCLC proliferation and metastasis, with a particular emphasis on its regulatory effects on APOE‐mediated cholesterol metabolism. By elucidating the molecular mechanism by which OVOL1 affects the progression of NSCLC through the cholesterol metabolism pathway, we can provide a novel therapeutic target for treating NSCLC by exploiting the vulnerability of cancer cells associated with metabolic reprogramming.

## 2 Material and Methods

### 2.1 Mice

BALB/c nude mice aged 4–6 weeks were acquired from Beijing Vital River Laboratory Animal Technology Co. Ltd. in Beijing, China. The mice utilised in this research were housed in a designated pathogen‐free animal facility at Xuzhou Medical University in China. The animal studies conducted adhered to the ethical standards established by the university's Experimental Animal Ethics Committee and received committee approval. These measures were taken to ensure that the welfare of the animals used in the study was protected and that the research was conducted in an ethical manner.

### 2.2 Cell Lines

NSCLC cell lines A549 and H1299 were purchased from Procell (Wuhan, China). A549 cells were cultured in DMEM, and h1299 cells were cultured in RPMI‐1640. Both types of media were supplemented with 10% FBS (Procell, Wuhan, China), 100 U/mL of penicillin and 100 mg/mL of streptomycin (PenStrep).

### 2.3 Clinical Samples and Immunohistochemical Staining

Samples from the Affiliated Hospital of Xuzhou Medical University were obtained for clinical analysis. The process of immunohistochemical (IHC) staining was carried out in accordance with established protocols [25]. Detailed information can be found in Table S1.

### 2.4 Construction of Plasmids, Lentivirus and Stable Cells

Plasmids and lentivirus were constructed by GeneChem Co. Ltd. (Shanghai, China). After transfection, screen with puromycin for 2 weeks.

### 2.5 Proliferation and Apoptosis Assay

Various techniques were used to evaluate the growth of cancer cells including Celigo cell counting assay, methyl thiazolyl tetrazolium (MTT) cell viability assay and plate clone formation assay. These assays helped to measure the growth and viability of the cells in response to different treatments. Additionally, the caspase 3/7 Assay and Annexin V‐APC/PI flow cytometry were utilised to identify apoptotic cells resulting from the treatments.

### 2.6 Transwell and Wound Healing Assays

In the experiments on cell migration, 5 × 10^4^ cells/200 μL were introduced into the upper chamber while 800 μL of culture medium with 10% serum were placed in the lower chamber. During the invasion evaluation, the top section was coated with Matrigel (Cat No. 356237; thinned at a proportion of 1:10; BD Biosciences, San Jose, CA, USA), and 5 × 10^4^ cells were added to the upper chamber in medium without serum. After 24 h of experiment, the samples were fixed with methanol and stained with crystal violet for 15 min.

### 2.7 Tumour Xenograft Study

A total of 20 mice were divided into two groups: shControl and shOVOL1, and then 5 × 10^6^ H1299 cells with or without OVOL1 knockdown were injected subcutaneously. Tumour volumes were measured weekly for 5 weeks before tissue harvest. Tumour volume (*V*) was computed using the formula: *V* = (length × width^2^)/2.

### 2.8 Lung Metastasis Model

Luciferase‐tagged H1299 cells (5 × 10^6^), with or without OVOL1 knockdown, were injected intravenously via the tail vein. After 4 weeks, bioluminescence was assessed, and lung tissues were harvested for analysis.

### 2.9 Liquid Chromatography Tandem Mass Spectrometry

Quantitative analysis was done by liquid chromatography tandem mass spectrometry as described previously [25].

### 2.10 Western Blot Analysis

Proteins were extracted using the Mammalian Protein Extraction Kit from CWBIO in China. After quantification of the extracted protein, protein immunoblotting was performed using sodium dodecyl sulfate polyacrylamide gel electrophoresis (SDS‐PAGE) and transferred. Seal with protein‐free fast blocking buffer for 15 min, and incubate the primary antibody and membrane overnight on a shaking bed at 4°C. Afterwards, incubate the secondary antibody at room temperature for 1.5 h, wash and test.

### 2.11 Quantitative Reverse Transcription Polymerase Chain Reaction Analysis

Fluorescence PCR tests were conducted in real‐time using the RT‐PCR Kit from CWBIO in China, with strict adherence to the specified procedures.

- OVOL1 Forward Primer: AAGAGACACGTCCGAACTCAC.
- OVOL1 Reverse Primer: TCCTCACACACGTACAGCTTG.
- APOE Forward Primer: GTTGCTGGTCACATTCCTGG.
- APOE Reverse Primer: GCAGGTAATCCCAAAAGCGAC.

### 2.12 Quantification and Statistical Analysis

The mean ± standard deviation (SD) data were reported. Statistical analyses were conducted with GraphPad Prism 8.0.2 (San Diego, CA, USA). Specific statistical analysis details are provided in the figure legends.

## 3 Results

### 3.1 OVOL1 Is Overexpressed in NSCLC and Correlated With Poor Prognosis in NSCLC Patients

**FIGURE 1:**
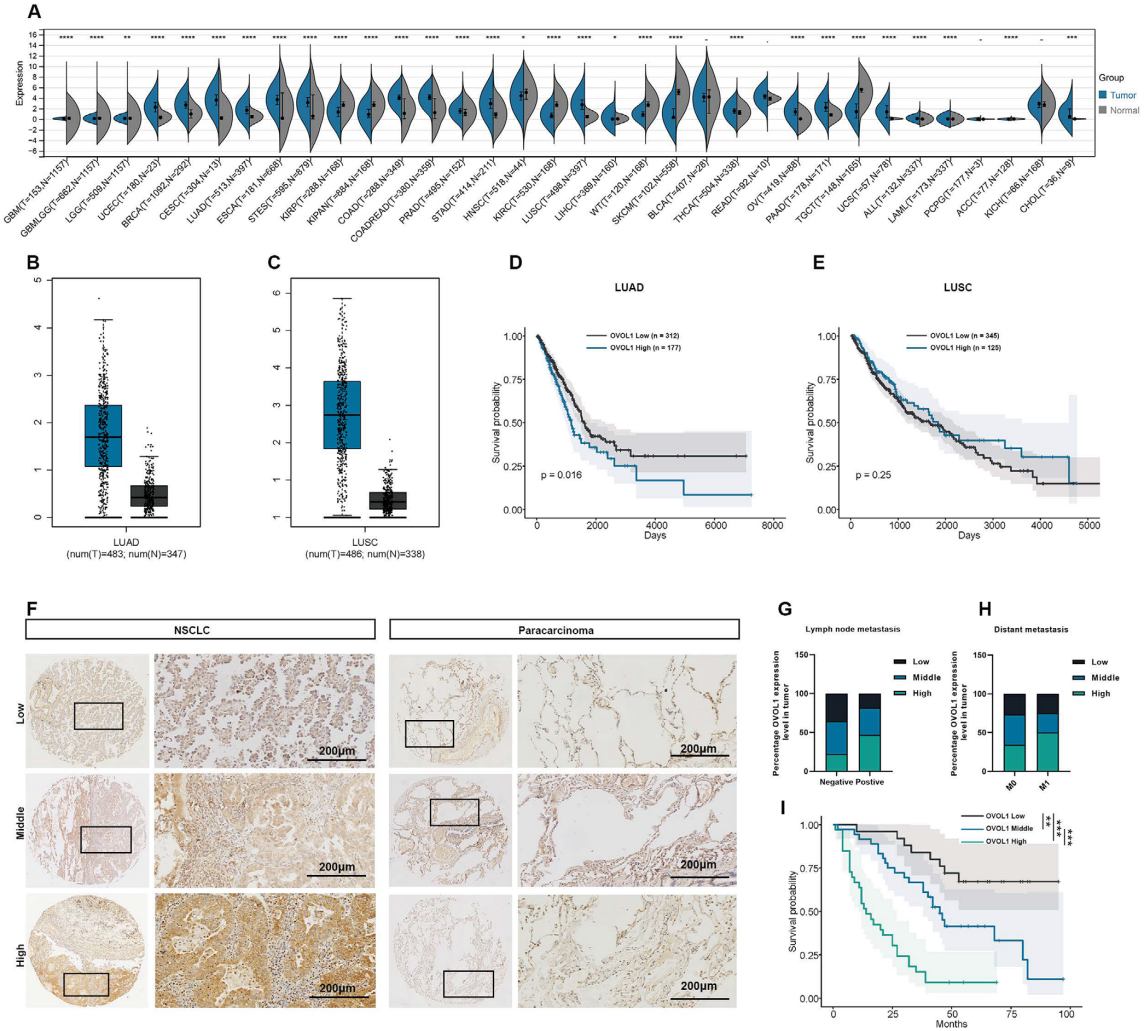
OVOL1 is upregulated in LUAD and associated with poor prognosis. (A) The OVOL1 expression in different human cancers from TCGA and GTEX database. (B, C) OVOL1 expression of LUAD and LUSC from GEPIA (http://gepia.cancerpku.cn/detail.php). (D, E) Kaplan–Meier survival curve analysis of LUAD and LUSC according to TCGA database. (F) Representative images of human NSCLC tumours and paracancerous tissue stained with OVOL1, bar = 200 μm. (G, H) OVOL1 is highly expressed in NSCLC and is associated with distant metastasis and lymph metastasis in 94 NSCLC patients, chi‐square test. (I) Kaplan–Meier survival curve analysis was performed to detect the effects of OVOL1 on the survival rate in NSCLC (*n* = 94), log‐rank test. *p < 0.05, **p < 0.01, ***p < 0.001.

To examine the expression levels of OVOL1 in different types of cancers, TCGA and GTeX datasets were analysed. OVOL1 expression was significantly higher in most malignant tumours, including lung adenocarcinoma (LUAD) and lung squamous cell carcinoma (LUSC) (Figure 1A–C). Subsequently, Kaplan—Meier survival analysis found that patients with elevated levels of OVOL1 experienced decreased overall survival and poorer prognosis, but there was no significant difference in LUSC patients (Figure 1D,E).

This research involved the examination of cancerous and neighbouring tissues in 94 individuals diagnosed with NSCLC. Immunohistochemical staining results indicated a notable increase in OVOL1 expression in tumour tissues compared to adjacent tissues (Figure 1F). Our study revealed a significant association between OVOL1 levels and lymph node metastasis and tumour stage in patients with non‐small cell lung cancer (NSCLC) (Figure 1G,H). Additionally, Kaplan–Meier survival analysis indicated that higher levels of OVOL1 were linked to a poorer prognosis in NSCLC patients (Figure 1I). These findings provide further support for the correlation between OVOL1 overexpression and reduced patient survival.

### 3.2 Knockdown of OVOL1 Inhibits NSCLC Cell Proliferation In Vitro and In Vivo

**FIGURE 2:**
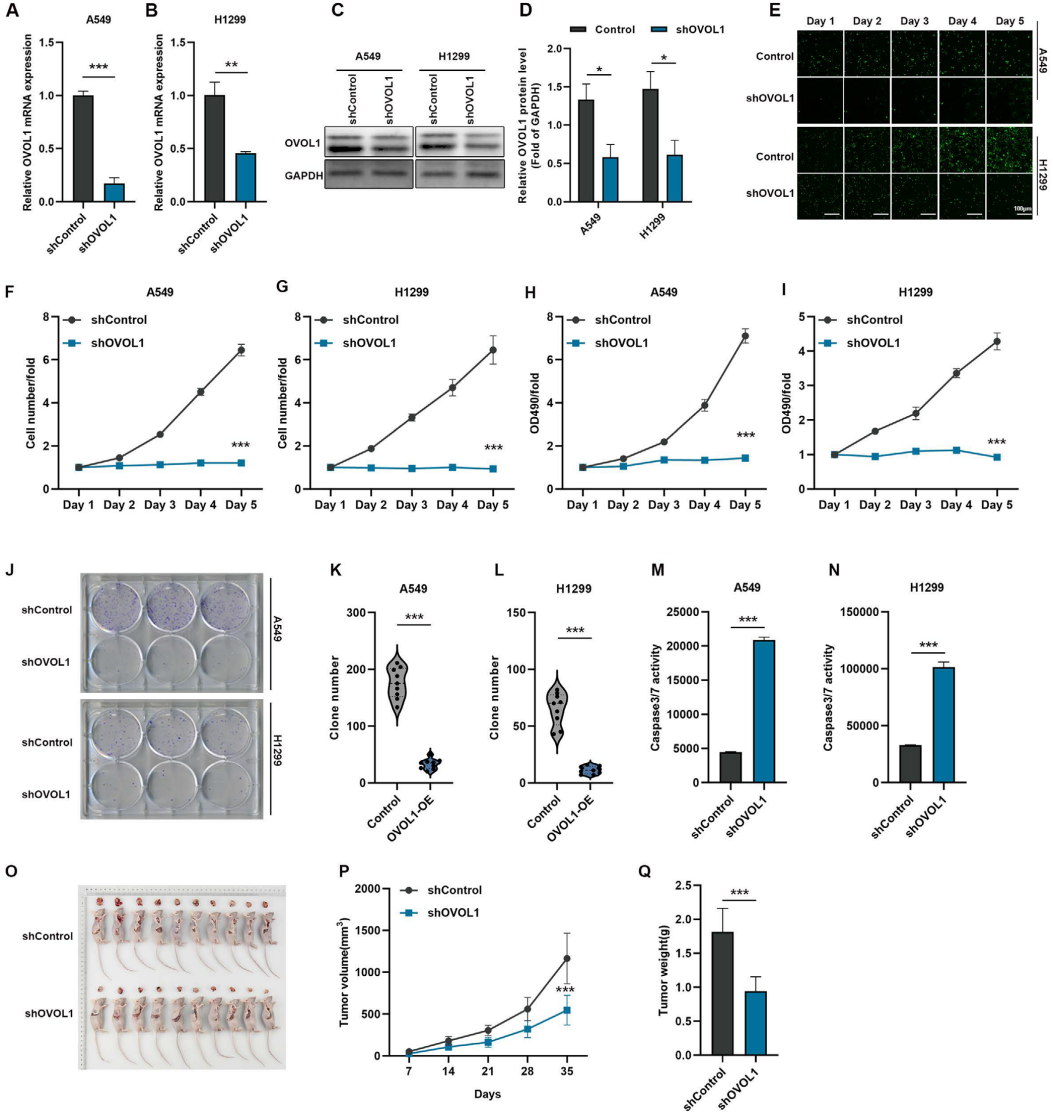
OVOL1 knockdown inhibits the proliferation of A549 and H1299 cells in vivo and vitro. (A–D) qRT‐PCR and western blot analysis of OVOL1 expression with or without OVOL1 knockdown in A549 and H1299 cells. (E) Representative images of Celigo cell counting assay, bar = 100 μm. (F, G) Quantitative statistics of Celigo cell counting assay in A549 and H1299 cells with or without OVOL1 knockdown. (H, I) MTT assay was used to determine the proliferation of H1299 and A549 cells with or without OVOL1 knockdown. (J–L) Colony formation assay for OVOL1 knockdown in A549 and H1299 cells with or without OVOL1 knockdown. (M, N) Caspase3/7 assay was used to detect the apoptosis in A549 and H1299 cells with OVOL1 knockdown. (O–Q) The effects of OVOL1 knockdown on tumour volume and weight are assessed using the mouse xenograft model (*n* = 10). *p < 0.05, **p < 0.01, ***p < 0.001 versus the shControl group, two‐tailed Student's *t*‐test.

To determine the function of OVOL1, we first screened NSCLC cell lines in our lab for OVOL1 expression. We selected A549 and H1299 cell lines as experimental cells (Figure S1). We constructed OVOL1 knockdown stable cell lines to further investigate its function in lung cancer cell lines. PCR and Western blot analysis revealed a notable decrease in OVOL1 expression in H1299 and A549 cells (Figure 2A–D). To delineate the influence of OVOL1 on the growth of A549 and H1299 cells, we conducted a series of assays. These included the Celigo cell counting assay (Figure 2E–G), the MTT cell viability assay (Figure 2H,I), and the plate clone formation assay (Figure 2J–L). Our data revealed that silencing OVOL1 markedly reduced the proliferation of these cancer cells. Furthermore, we assessed the impact of reduced OVOL1 expression on cellular apoptosis by measuring caspase‐3/7 activity and flow cytometry, and findings confirmed that OVOL1 depletion escalated apoptosis in both A549 and H1299 cell lines (Figure 2M,N). Furthermore, the mouse xenograft model showed that H1299 with shOVOL1 treatment significantly reduced tumour weight and volume (Figure 2O–Q; Figure S2).

### 3.3 Knockout of OVOL1 Suppresses Lung Cancer Metastasis In Vitro and In Vivo

**FIGURE 3:**
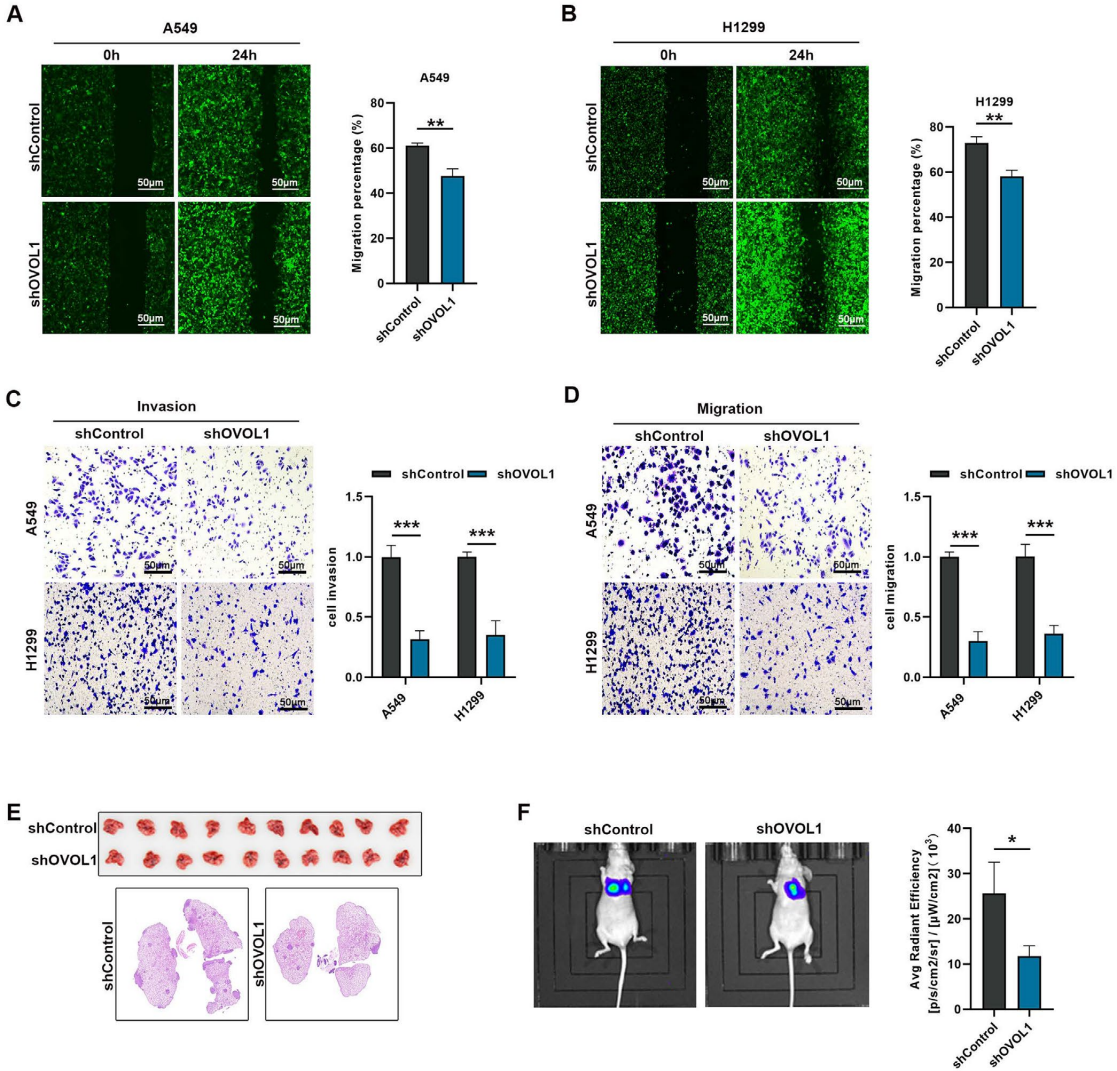
OVOL1 knockdown suppress lung cancer metastasis in vivo and vitro. (A, B) Representative image of scratch assay in A549 and H1299 cells with or without OVOL1 knockdown (*n* = 3), bar = 100 μm. (C, D) Representative images of cell migration and invasion assays and quantitative. Data in A549 and H1299 cells with or without OVOL1 knockdown (*n* = 3), bar = 50 μm. (E) OVOL1 knockdown decreases lung tumour burden in mice 4 weeks after i.v. injection of H1299 cells (*n* = 10). (F) Representative image of bioluminescence with or without OVOL1 knockdown. *p < 0.05, **p < 0.01, ***p < 0.001 versus the shControl group, two‐tailed Student's *t*‐test.

We next investigated the migration of A549 and H1299 cells with or without OVOL1 knockdown utilising wound healing assays (Figure 3A,B) and Transwell assays (Figure 3C,D). Data from these approaches indicated a reduction in migration and invasion capabilities of NSCLC cells upon OVOL1 silencing. To extend these findings to an in vivo setting, H1299 cells labelled with luciferase were injected into Balb/c nude mice. Following histological examination of the lung tissue, it was confirmed that suppressing OVOL1 significantly decreased the spread of H1299 cells to the lungs and decreased the size of the original tumour (Figure 3E). The luciferase reporter gene assay corroborated the histological assessment, showing congruent results (Figure 3F).

### 3.4 Proteomic Analysis of H1299 Cells With OVOL1 Knockdown Reveals Upregulation of Cholesterol Metabolism

**FIGURE 4:**
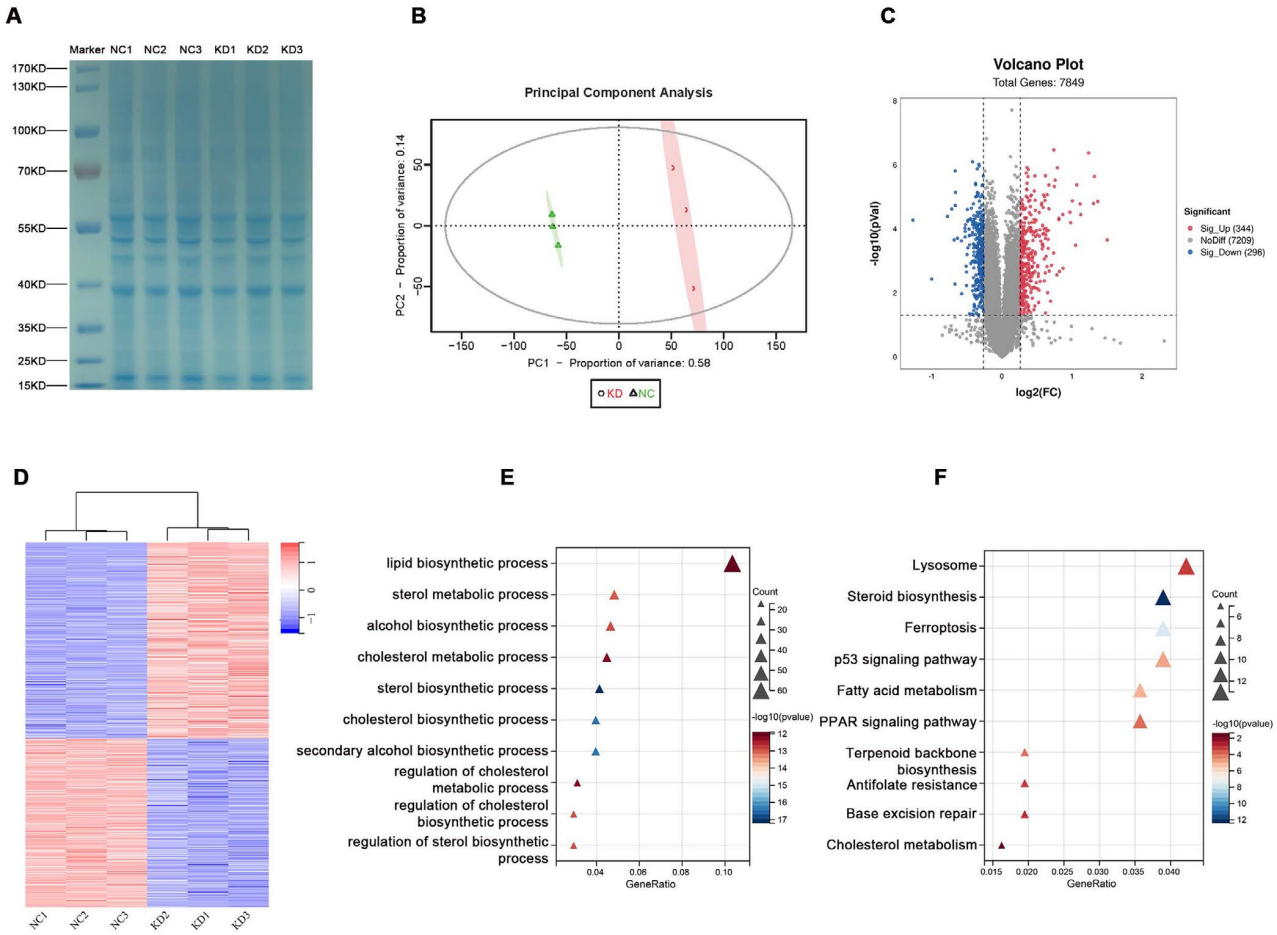
Proteomic profiling of H1299 cells with OVOL1 knockdown. (A) Coomassie brilliant blue staining of proteins immunoprecipitated with anti‐OVOL1 antibody from total cell lysates of H1299 cells. (B) PCA scores plot for H1299 cells with or without OVOL1 knockdown. (C) Volcano plot of the differential gene expression distribution. (D) Hierarchical clustering results for proteins in shControl versus shOVOL1 group. (E) GO term enrichment analysis of significant proteins. (F) KEGG pathway enrichment analysis of significant proteins.

We next attempted to determine the potential mechanism by which OVOL1 regulates lung cancer proliferation and invasion. We conducted proteomic analysis on H1299 cells with or without OVOL1 knockdown and identified 7849 proteins. Results from the SDS‐PAGE and PCA indicate that the level of similarity within groups and the extent of variation between groups fall within acceptable ranges (Figure 4A,B). In addition, our analysis showed an increase in 344 genes and a decrease in 296 genes in the shOVOL1 group compared to the control group (Figure 4C,D). We also assessed gene ontology (GO) and Kyoto Gene and Genome Encyclopedia pathway enrichment analyses of these genes, which showed functional enrichment of lipid biosynthetic process, cholesterol metabolic process, cholesterol biosynthetic process, fatty acid metabolism, and cholesterol metabolism in the shOVOL1 group relative to controls (Figure 4E,F).

### 3.5 OVOL1 Promotes NSCLC Progression by Enhancing Cholesterol Metabolism

**FIGURE 5:**
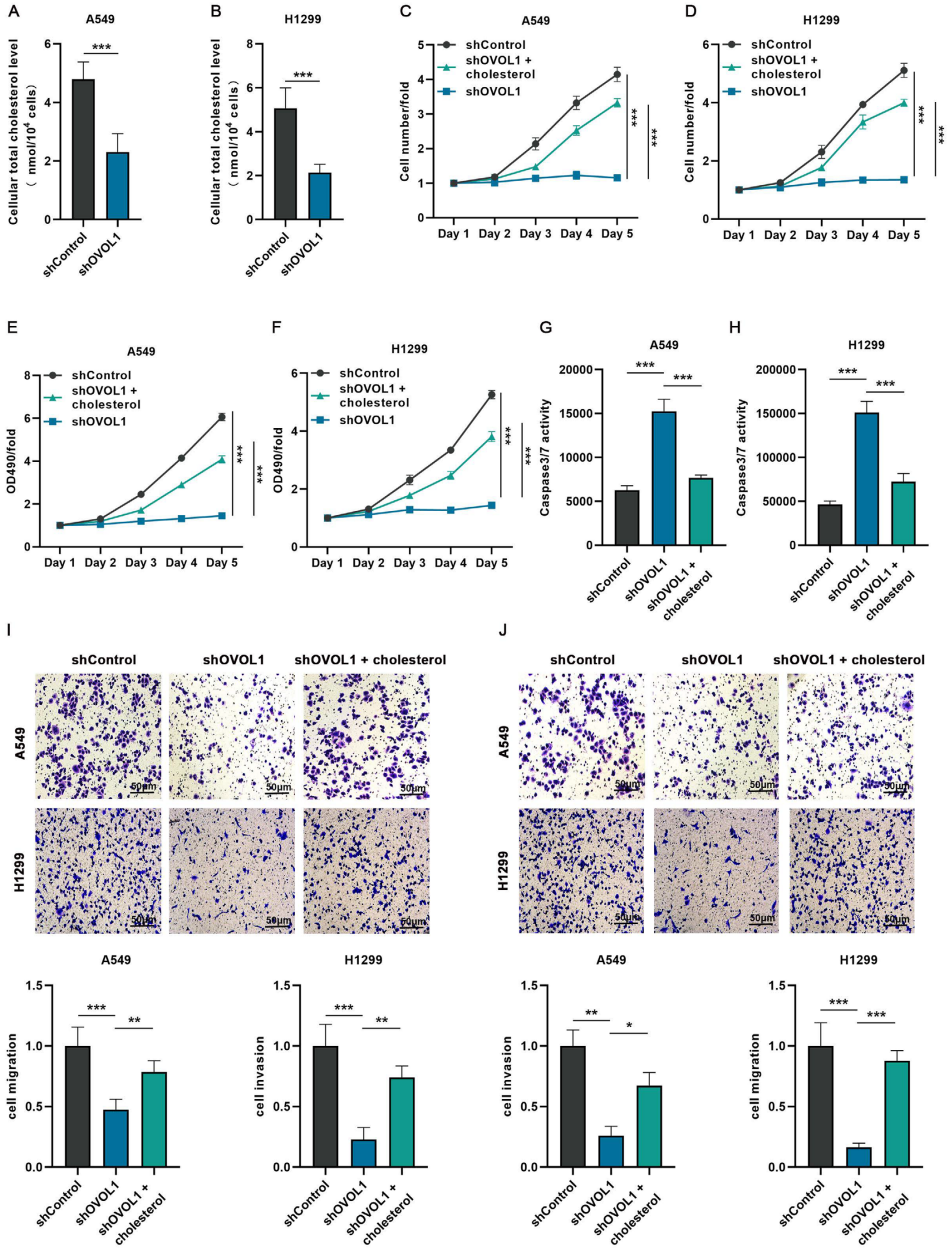
Cholesterol promotes proliferation and metastasis of A549 and H1299 cells. (A, B) Total cholesterol kit for detecting intracellular cholesterol in A549 and H1299 cells with shOVOL1 treatment. (C, D) Quantitative statistics of Celigo cell counting assay in A549 and H1299 cells with or without Cholesterol treatment. (E, F) MTT assay was used to determine the proliferation of H1299 and A549 cells with or without Cholesterol treatment. (G, H) Caspase3/7 assay was used to detect the apoptosis in A549 and H1299 cells with Cholesterol treatment. (I, J) Representative images of cell migration and invasion assays and quantitative data in A549 and H1299 cells with or without Cholesterol treatment (*n* = 3), bar = 50 μm. *p < 0.05, **p < 0.01, ***p < 0.001 versus the indicated group, one‐way ANOVA.

We measured intracellular cholesterol content. The results showed that intracellular cholesterol significantly decreased after OVOL1 knockdown (Figure 5A,B). Results from the Celigo cell count assay and MTT cell viability assay indicated that cholesterol treatment hindered the impact of OVOL1 knockdown on NSCLC cell growth (Figure 5C–F). In addition, the impact of cholesterol treatment on apoptosis was assessed through caspase 3/7 activity assay and flow cytometry, and the results showed that cholesterol treatment inhibited OVOL1 knockdown on apoptosis of A549 and H1299 cells (Figure 5G,H; Figure S3). Next, we used the Transwell assay to explore the migration and invasion of A549 and H1299 cells treated with cholesterol after OVOL1 knockdown, showing that cholesterol treatment promotes tumour cell invasion and migration (Figure 5I,J). These results demonstrate that OVOL1 knockdown inhibits proliferation, migration and invasion of NSCLC cells by reducing cholesterol metabolism.

### 3.6 OVOL1 Promotes Cholesterol Metabolism by Upregulating APOE Expression

**FIGURE 6:**
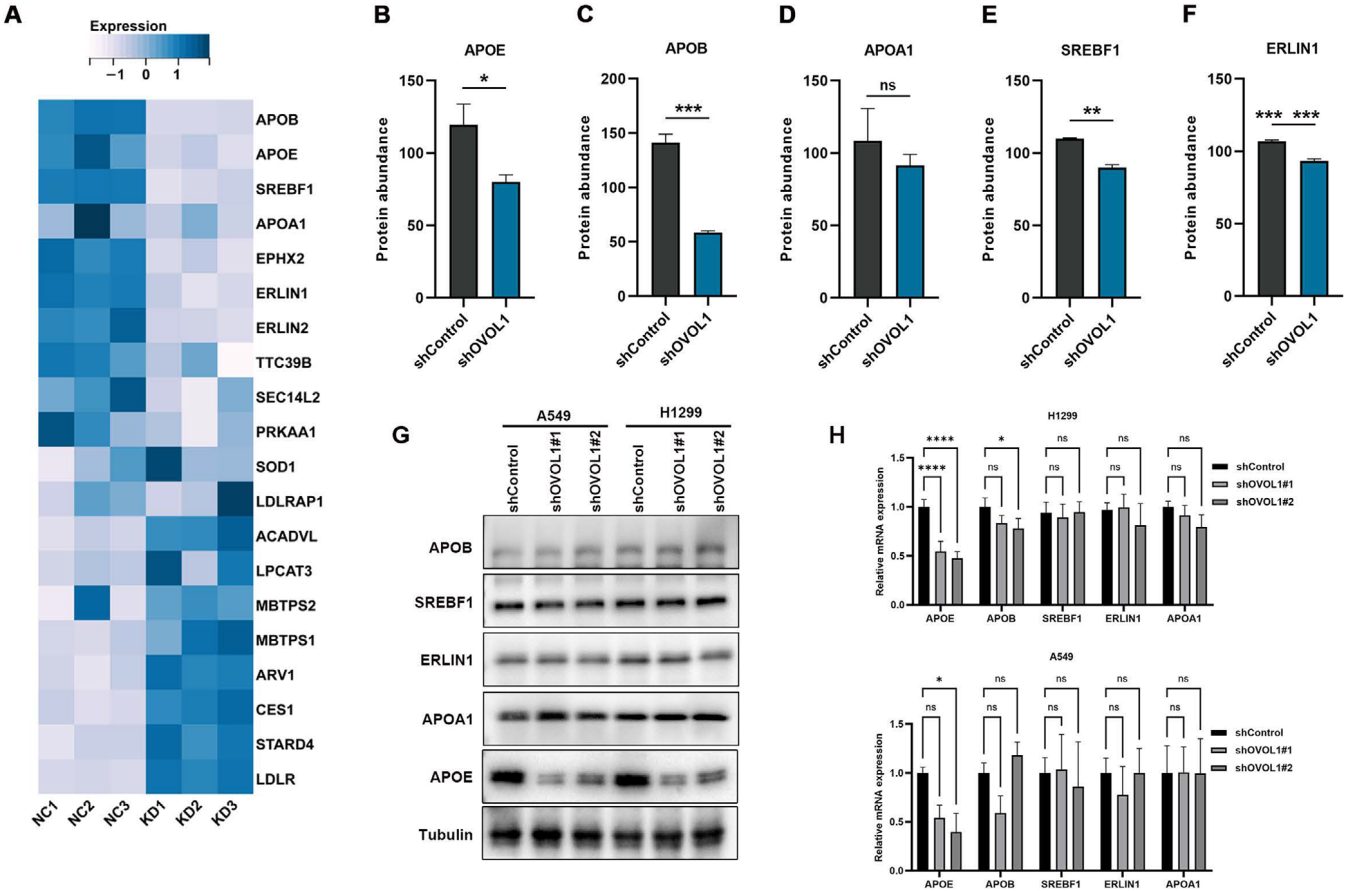
APOE is a potential downstream target of OVOL1. (A) Heatmap of cholesterol metabolism. (B–F) Expression levels of 10 candidate genes for cholesterol metabolism in TMT label‐based LC–MS/MS and PRM validation (*n* = 3). (G, H) Western blot analysis of APOE expression with shOVOL1 treatment. *p < 0.05, **p < 0.01, ***p < 0.001 versus the indicated group, one‐way ANOVA.

Proteomic analysis showed that the lung cancer process regulated by OVOL1 is strongly associated with cholesterol metabolism. Therefore, we selected 5 proteins related to cholesterol metabolism from this differentially expressed protein. The results showed that the levels of APOE, APOB, SREBF1 and ERLIN1 were regulated by OVOL1 knockdown (Figure 6A–F). Notably, APOE plays a crucial role in the transport of cholesterol across cell membranes. PCR and Western blot experiments further confirmed that APOE is regulated by OVOL1 (Figure 6G,H).

**FIGURE 7:**
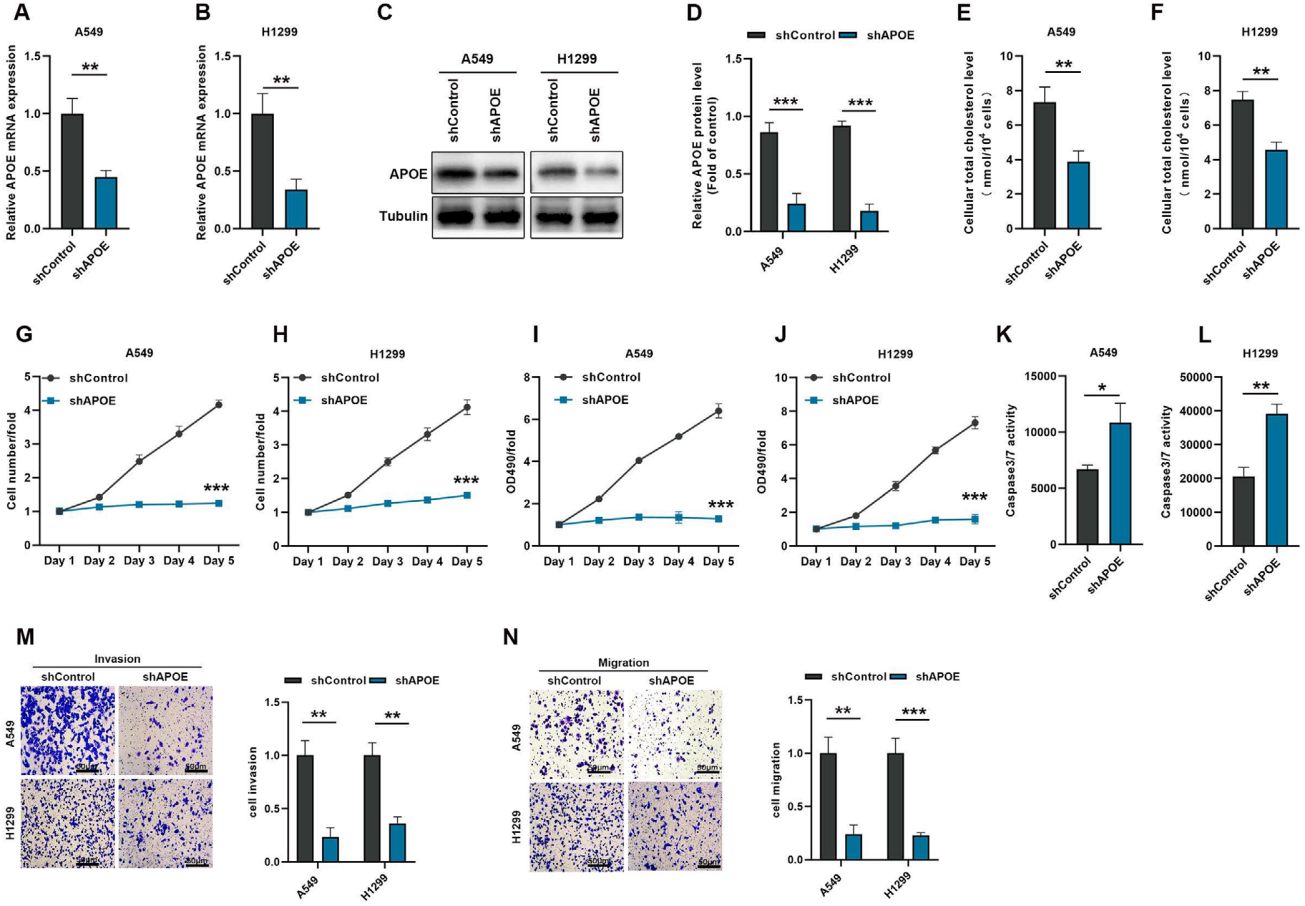
APOE knockdown inhibits cholesterol uptake, proliferation and metastasis in A549 and H1299 cells. (A–D) Determination of APOE mRNA and protein level with shAPOE treatment. (E, F) Total cholesterol kit for detecting intracellular cholesterol in A549 and H199 cells with shAPOE treatment. (G, H) Quantitative statistics of Celigo cell counting assay in A549 and H1299 cells with or without shAPOE treatment. (I, J) MTT assay was used to determine the proliferation of H1299 and A549 cells with or without shAPOE l treatment. (K, L) Caspase3/7 assay was used to detect the apoptosis in A549 and H1299 cells with shAPOE treatment. (M, N) Representative images of cell migration and invasion assays and quantitative. Data in A549 and H1299 cells with or without shAPOE treatment (*n* = 3), bar = 50 μm. *p < 0.05, **p < 0.01, ***p < 0.001 versus the shControl group, two‐tailed Student's *t*‐test.

To examine the effects of APOE on cholesterol uptake, migration and invasion in NSCLC cells, we constructed APOE‐knockdown A549 and H1299 stable cell lines (Figure 7A–D). Total cholesterol assay showed a significant reduction in intracellular cholesterol content after APOE knockdown (Figure 7E,F). In addition, APOE knockdown also showed decreased proliferation, invasion and migration capacity, and increased apoptotic activity (Figure 7G–N; Figure S4). In conclusion, the above data suggest that APOE‐mediated cholesterol uptake plays an important role in the process of OVOL1‐regulated NSCLC process.

### 3.7 APOE‐Mediated Cholesterol Metabolism Is Required for OVOL1 Carcinogenesis

**FIGURE 8:**
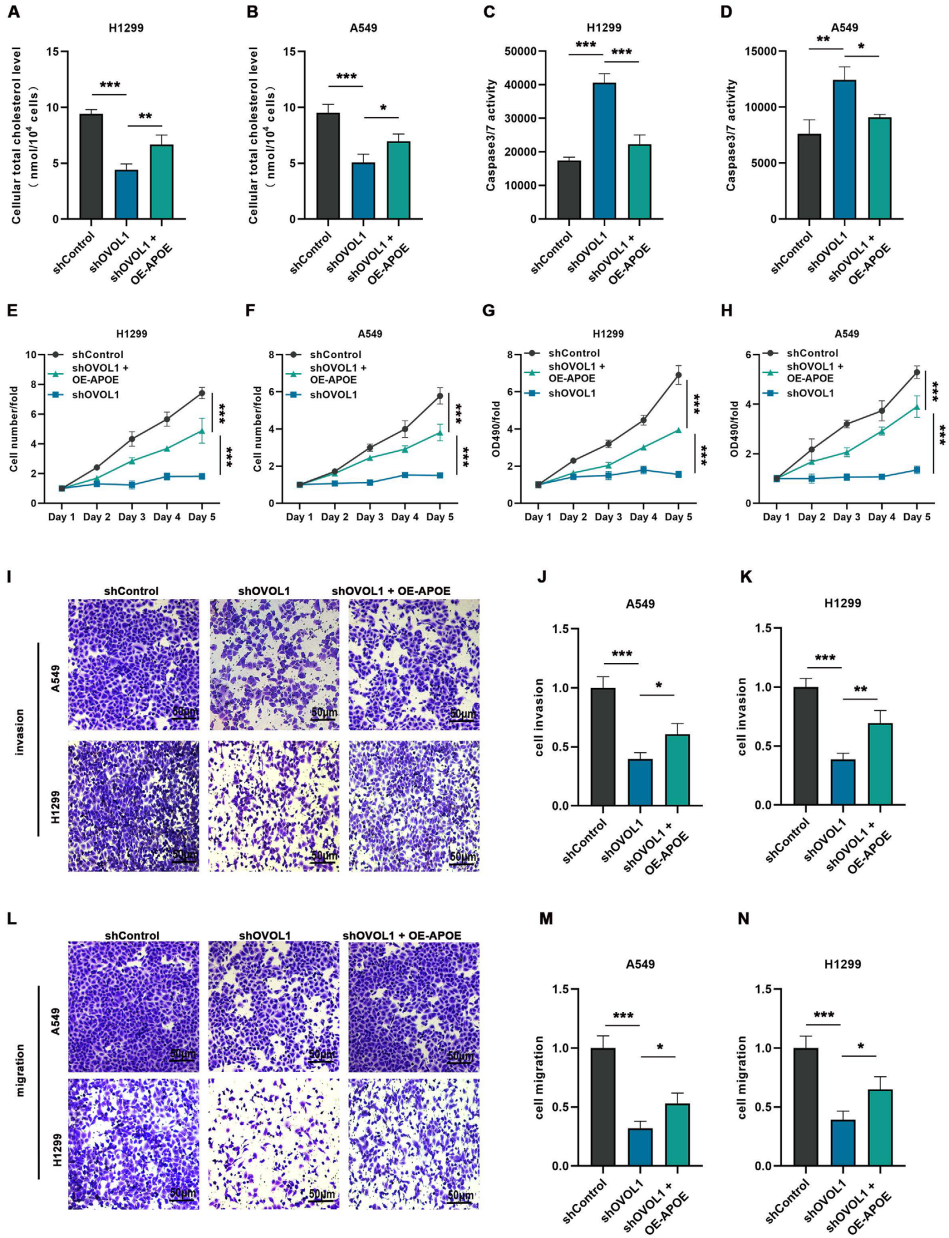
APOE contributes to the overexpression of OVOL1 carcinogenic effects in vitro and in vivo. (A, B) Overexpression of APOE after OVOL1 knockdown rescue the cholesterol uptake of H1299 and A549 cells. (C, D) Caspase3/7 was used to detect the apoptotic activity of OVOL1‐knockdown A549 and H1299 with or without overexpression of APOE. (E–H) CCK‐8 and Celigo assays were used to determine the rescue effect of APOE overexpression on OVOL1 knockdown‐mediated inhibition of proliferation in H1299 and A549 cells. (I–N) Transwell measured the salvage effect of APOE overexpression on OVOL1 knockdown mediated invasion and migration inhibition in H1299 and A549 cells. *p < 0.05, **p < 0.01, ***p < 0.001 versus the shControl group, one‐way ANOVA.

To evaluate the role of APOE in OVOL1‐induced NSCLC progression, we overexpressed APOE in OVOL1‐knockdown H1299 and A549 cells. Assessment of total cholesterol demonstrated that overexpression of APOE partly reversed the reduction in intracellular cholesterol levels instigated by the knockdown of OVOL1 (Figure 8A,B). Moreover, our results indicated that APOE overexpression mitigated the enhancement in apoptosis (Figure 8C,D; Figure S5) and the suppression of both cell proliferation (Figure 8E,H), invasion and migration (Figure 8I–N) in NSCLC cells attributed to OVOL1 knockdown. Collectively, these findings imply that APOE significantly contributes to the progression of NSCLC as modulated by OVOL1.

## 4 Discussion

In this study, the TCGA database and clinical specimen information analysis showed that elevated OVOL1 expression correlated with diminished survival prospects in NSCLC patients. High expression of OVOL1 in NSCLC enhanced a variety of tumour features, including proliferation and metastasis. Furthermore, our findings indicated that OVOL1 promoted cholesterol metabolism by increasing the expression levels of APOE. By delving into the molecular interlinkages between cholesterol metabolic pathways and carcinogenic processes, our findings could be targets for therapeutic interventions in NSCLC.

OVOL1 plays a pivotal role in the maturation and differentiation of epithelial lineages across diverse tissues, such as skin, kidney and mammary epithelium [26, 27, 28, 29, 30, 31]. Previous research has documented OVOL1's contributions to hair follicle development, spermatogenesis and renal maturation in murine models [29]. Operating downstream of the Wnt/β‐catenin signalling cascade in both mice and Drosophila [32], OVOL1 has also been implicated in the suppression of atopic dermatitis through modulation of FLG expression in mouse keratinocytes [33]. Furthermore, OVOL1 has demonstrated an inhibitory effect on breast cancer progression by enhancing the degradation of TGF‐β type I receptors [34]. The axis has been identified as a regulator of the proto‐oncogene C‐MYC, influencing the progression from orthotopic epidermal malignancy (Bowen disease) to invasive squamous cell carcinoma [35]. Despite these insights, the involvement of OVOL1 in lung cancer remains poorly understood. Our studies have further elucidated the role of OVOL1 in NSCLC. Specifically, we observed that OVOL1 knockdown inhibited the proliferative, invasive and migratory capabilities of NSCLC cells in vitro. Complementary in vivo experiments corroborated these findings, revealing that OVOL1 knockout inhibited the proliferation and metastasis of NSCLC. These findings suggest that OVOL1 is a promising prognostic and therapeutic target for patients with NSCLC.

Cholesterol metabolism is a crucial characteristic of malignant tumours. Numerous studies have confirmed the efficacy of anti‐tumour therapies targeting cholesterol metabolic pathways. As rapidly proliferating cells, cancer cells require elevated levels of cholesterol to support membrane biogenesis and other functional needs. Cholesterol metabolism plays a significant role in cancer progression, including cell proliferation, migration and invasion. Tumours with rapid proliferation facilitate cancer progression by enhancing cholesterol biosynthesis and increasing exogenous cholesterol uptake. Statins and HMGCR inhibitors have been the most widely researched cholesterol metabolism‐targeted drugs in clinical studies involving cancer patients. Extensive clinical research has demonstrated that statin use is beneficial for patient survival. A large‐scale clinical study found that patients treated with statins had a 47% lower risk of developing colorectal cancer compared to those who did not use statins. Additionally, another study showed that statins reduced mortality in patients with various cancer types, regardless of whether the statins were taken before or after a cancer diagnosis. Consequently, cholesterol depletion or transport blockade impedes tumour growth and invasion in various cancers. Our study demonstrated that knockdown of OVOL1 leads to impaired cholesterol metabolism in lung cancer cells. Mechanistically, OVOL1 knockout reduces cholesterol uptake by APOE.

Indeed, APOE is pivotal in the transport of lipids and the metabolism of lipoproteins [36, 37]. Furthermore, APOE partakes in an array of biological processes related to tumour progression, such as the augmentation of tumour cell growth, the initiation of metastasis, and the promotion of angiogenesis [38, 39]. Previous studies have shown that APOE enhances the proliferation and growth of thyroid cancer cells by promoting cellular glycolysis [40]. In gastric cancer, tumour‐associated macrophages (TAMs) contribute to tumour cell metastasis by secreting exosomes laden with APOE [41]. Furthermore, APOE has been shown to enhance the migration and invasion capabilities of colorectal cancer cells by interacting with low‐density lipoprotein receptor‐associated protein 1 (LRP1) [42]. Our research has revealed that the downregulation of OVOL1 results in diminished APOE expression both in mRNA and protein levels, which consequently decreases cholesterol uptake by the tumour cells. Additionally, our findings indicate that the absence of APOE prevented the proliferative capacity of the cells and mitigates the invasiveness and migratory propensity of non‐small cell lung cancer cells. Our analyses indicate that the transcription factor OVOL1 does not directly bind to the APOE gene promoter. This suggests that OVOL1 may regulate APOE expression indirectly, potentially through intermediary factors or signalling pathways, rather than through direct protein‐DNA interactions. However, we lack direct evidence of an interaction between OVOL1 and APOE or any potential intermediaries. Therefore, further studies are needed to elucidate the exact mechanism by which OVOL1 regulates APOE expression.

In conclusion, our investigation elucidates the pivotal role of OVOL1 in modulating the proliferation and metastasis of non‐small cell lung cancer (NSCLC) via its governance on APOE‐mediated cholesterol metabolic pathways. This revelation extends the current comprehension of the pathobiological underpinnings of NSCLC and concurrently heralds novel prospects for therapeutic intervention. The ensuing challenge will be to adeptly convert these molecular insights into effective clinical modalities, thereby enhancing the prognostic outcomes and augmenting the quality of life for individuals afflicted with NSCLC.

## Author Contributions

**Shoujie Feng:** funding acquisition (equal), resources (equal), software (equal), writing – original draft (equal), writing – review and editing (equal). **Li Zhang:** methodology (equal), project administration (equal), writing – original draft (equal), writing – review and editing (equal). **Teng Sun:** conceptualization (equal), data curation (equal), formal analysis (equal). **Lei Xu:** data curation (equal), investigation (equal), visualization (equal). **Xiaoyu Quan:** data curation (equal), formal analysis (equal). **Guoqing Zhao:** data curation (equal), formal analysis (equal), software (equal). **Hao Zhang:** validation (equal), visualization (equal).

## Consent

All authors have agreed to publish this manuscript.

## Conflicts of Interest

The authors declare no conflicts of interest.

## Supporting information


Figure S1.

Table S1.

Table S2.

## Data Availability

The datasets supporting the findings of this study are available in the TCGA (https://gdc.xenahubs.net) and GEO (https://www.ncbi.nlm.nih.gov/geo/) databases. The proteome data are available in Table S2.
