# Supplementary material for: OVOL1 Promotes Proliferation and Metastasis of Non‐Small Cell Lung Cancer by Regulating APOE‐Mediated Cholesterol Metabolism
Source: J Cell Mol Med. 2025 May 28;29(11):e70634. doi: 10.1111/jcmm.70634 (PMC12119240; doi:10.1111/jcmm.70634)
Supplement: Supplementary file 1 — Figure S1. [file JCMM-29-e70634-s001.docx]

**Supplementary Figures**


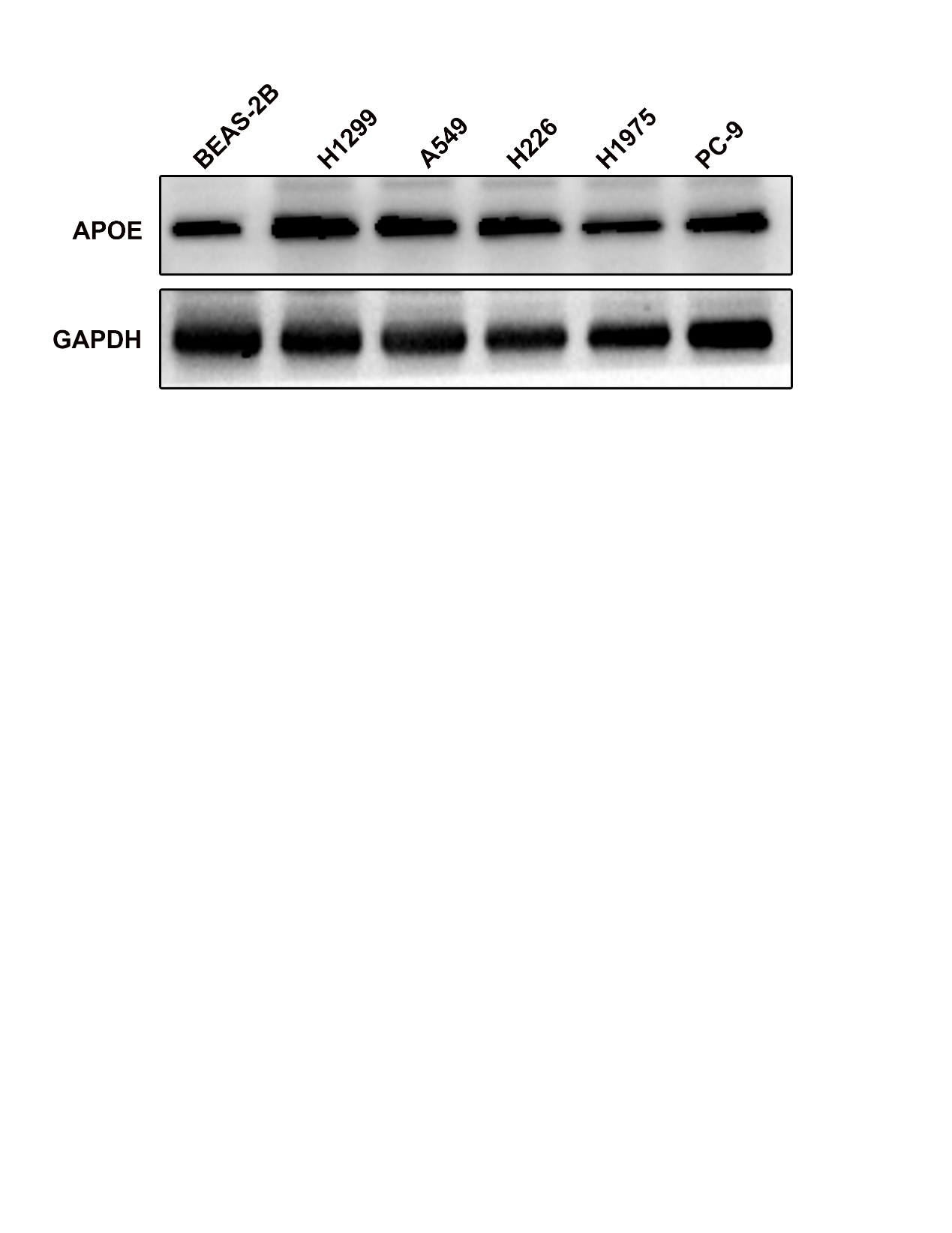


**Figure S1. Basal expression levels of OVOL1 in different lung cancer cells.**

(A) Western blot was used to detect the expression level of OVOL1 in BEAS-2B, H1299, A549, H226, H1975 and PC-9 cells.


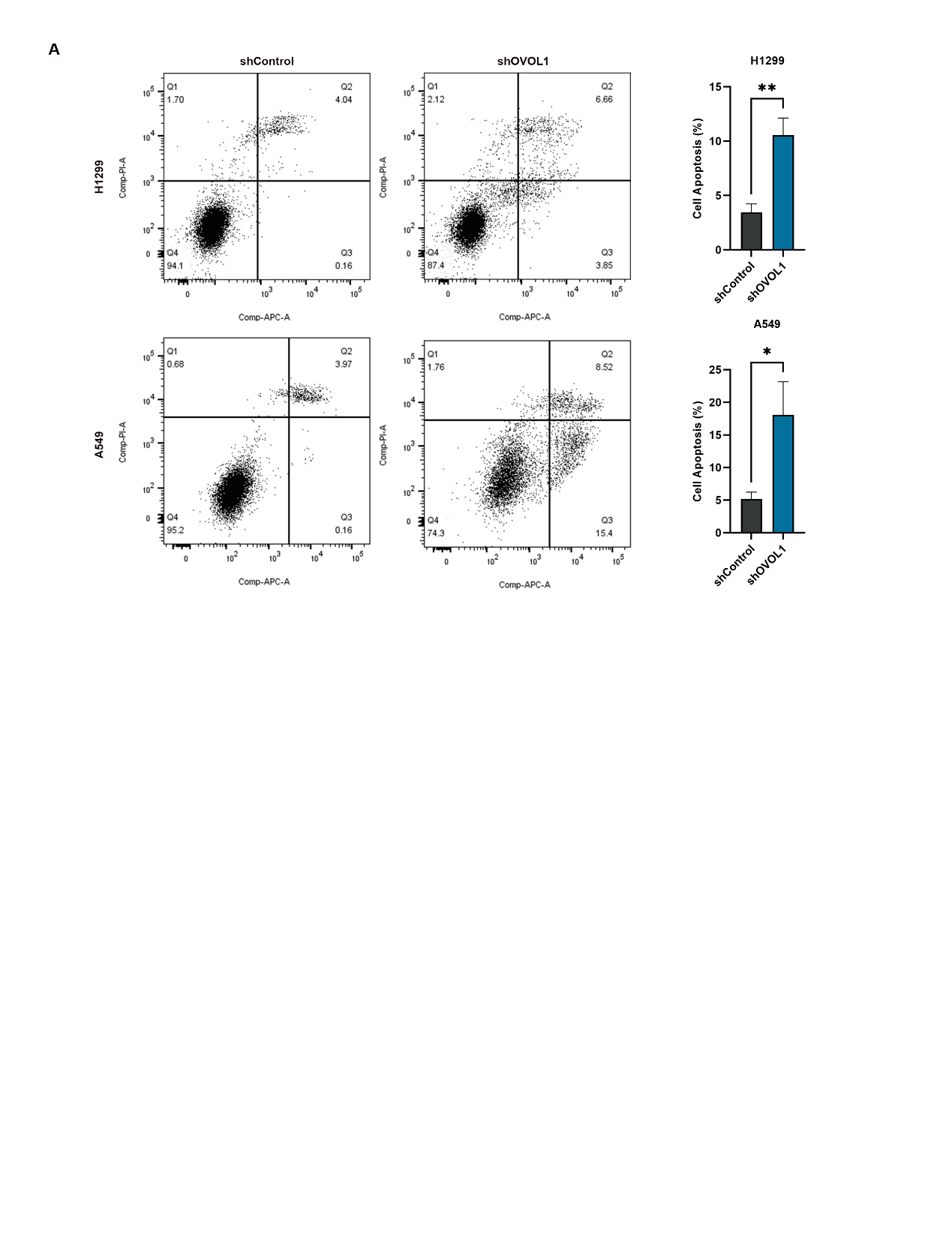


**Figure S2. Effect of OVOL1 knockdown on apoptosis of lung cancer cells.**

(A) Flow cytometry was used to detect the effect of OVOL1 knockdown on apoptosis of H1299 and A549 cells.


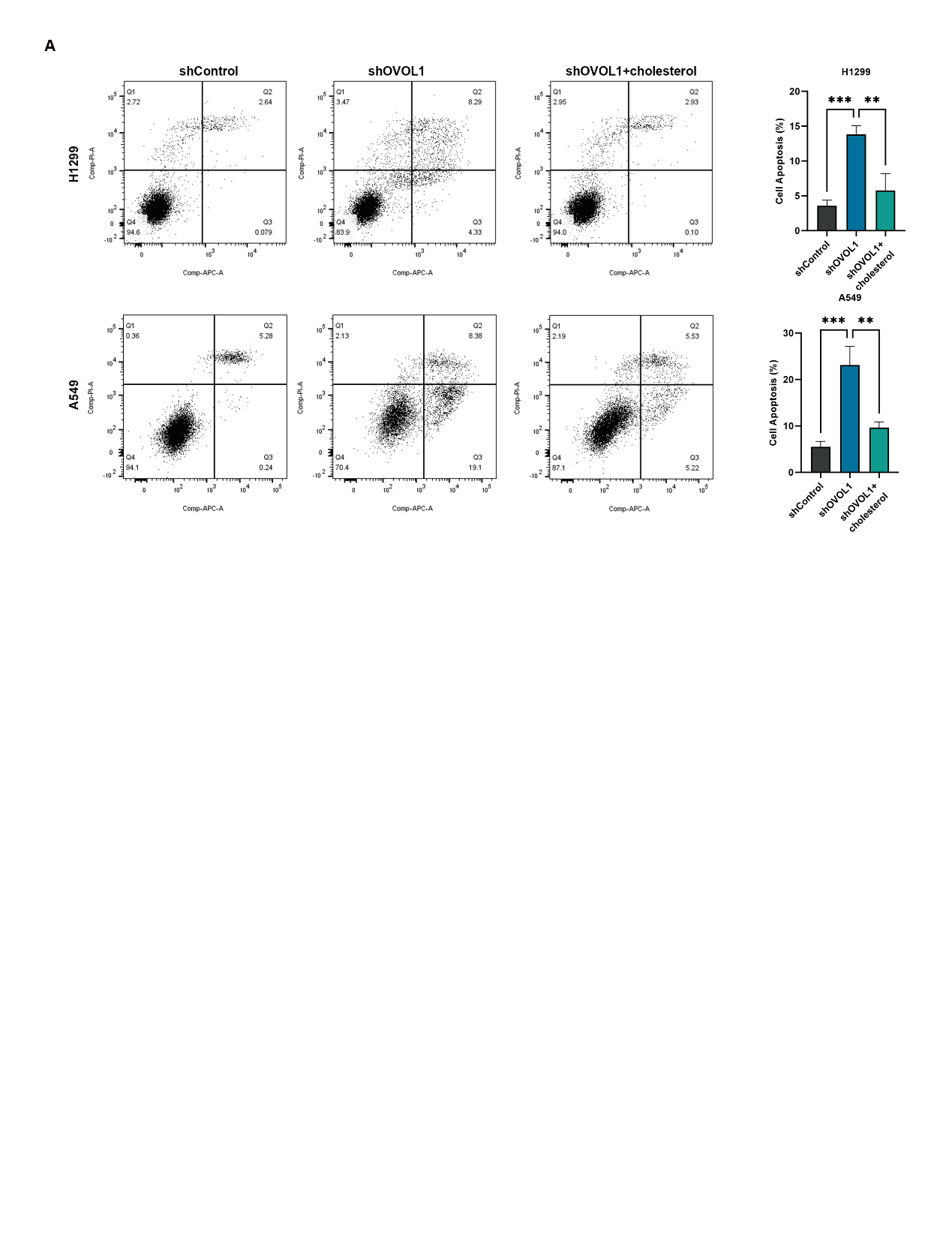


**Figure S3. Effect of cholesterol on apoptosis of lung cancer cells.**

(A) Flow cytometry was used to detect the effect of cholesterol addition on apoptosis of tumor cells with OVOL1 knockdown.


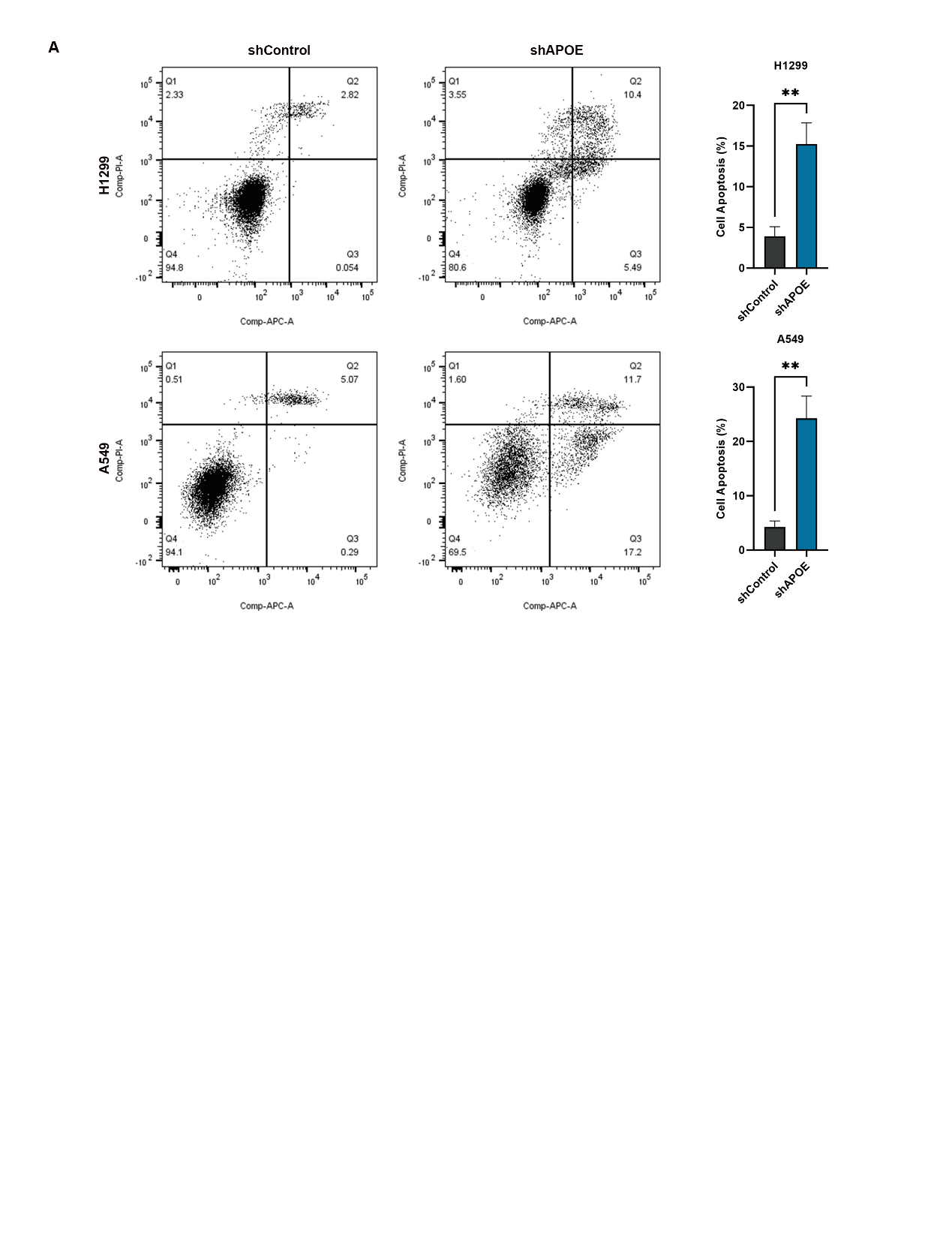


**Figure S4. Effect of APOE knockdown on apoptosis of lung cancer cells.**

(A) Flow cytometry was used to detect the effect of APOE knockdown on apoptosis of H1299 and A549 cells.


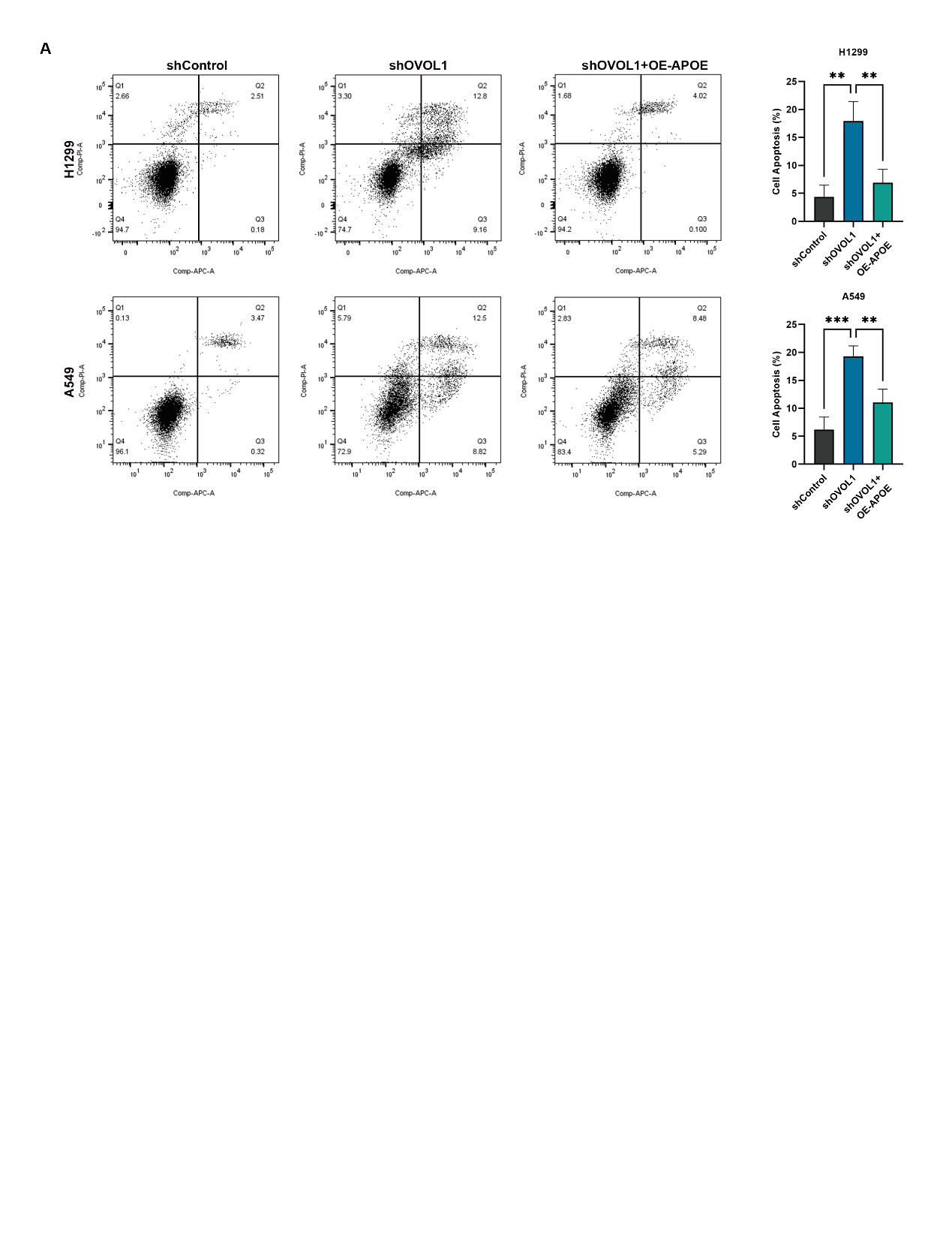


**Figure S5. Effect of APOE overexpression on apoptosis of lung cancer cells with OVOL1 knockdown.**

(A) Flow cytometry was used to detect the effect of APOE overexpression on apoptosis of tumor cells with OVOL1 knockdown.
