# Supplementary material for: OVOL1 Promotes Proliferation and Metastasis of Non‐Small Cell Lung Cancer by Regulating APOE‐Mediated Cholesterol Metabolism
Source: J Cell Mol Med. 2025 May 28;29(11):e70634. doi: 10.1111/jcmm.70634 (PMC12119240; doi:10.1111/jcmm.70634)
Supplement: Supplementary file 2 — Table S1. [file JCMM-29-e70634-s003.docx]

**Supplementary table 1 : Relationships between OVOL1 expression and the clinicopathological characteristics of NSCLC patients**

| **Variables** | **OVOL1 staining** | | | **Total(N=94)** | **P value** |
| --- | --- | --- | --- | --- | --- |
|  | **High(N=33)** | **Middle(N=36)** | **Low(N=25)** |  |  |
| **Age** |  |  |  |  |  |
| Mean±SD | 64.97±10.65 | 61.64±10.91 | 64.24±7.26 | 63.50±9.98 |  |
| Median[min-max] | 63.00[35.00,86.00] | 62.00[40.00,80.00] | 63.00[52.00,76.00] | 63.00[35.00,86.00] |  |
| **Gender** |  |  |  |  | 0.69 |
| Female | 14(33.33%) | 15(35.72%) | 13(30.95%) | 42(44.68%) |  |
| Male | 19(36.54%) | 21(40.38%) | 12(23.09%) | 52(55.32%) |  |
| **Pathologic type** | |  |  |  |  |
| LUAD | 33(35.11%) | 36(38.30%) | 25(26.60%) | 94(100.00%) |  |
| **Lymph node metastasis** | |  |  |  | 0.03 |
| Negative | 10(22.22%) | 19(42.22%) | 16(35.56%) | 45(47.87%) |  |
| Positive | 23(46.94%) | 17(34.69%) | 9(18.37%) | 49(52.13%) |  |
| **Distant metastasis** | |  |  |  | 0.79 |
| M0 | 31(34.44%) | 35(38.89%) | 24(26.67%) | 90(95.74%) |  |
| M1 | 2(50.00%) | 1(25.00%) | 1(25.00%) | 4(4.26%) |  |
| **Tumor stage** | |  |  |  | 0.02 |
| III~IV | 16(53.33%) | 12(40.00%) | 2(6.67%) | 30(31.91%) |  |
| II~III | 3(37.50%) | 3(37.50%) | 2(25.00%) | 8(8.51%) |  |
| I~II | 14(25.00%) | 21(37.50%) | 21(37.50%) | 56(59.57%) |  |
